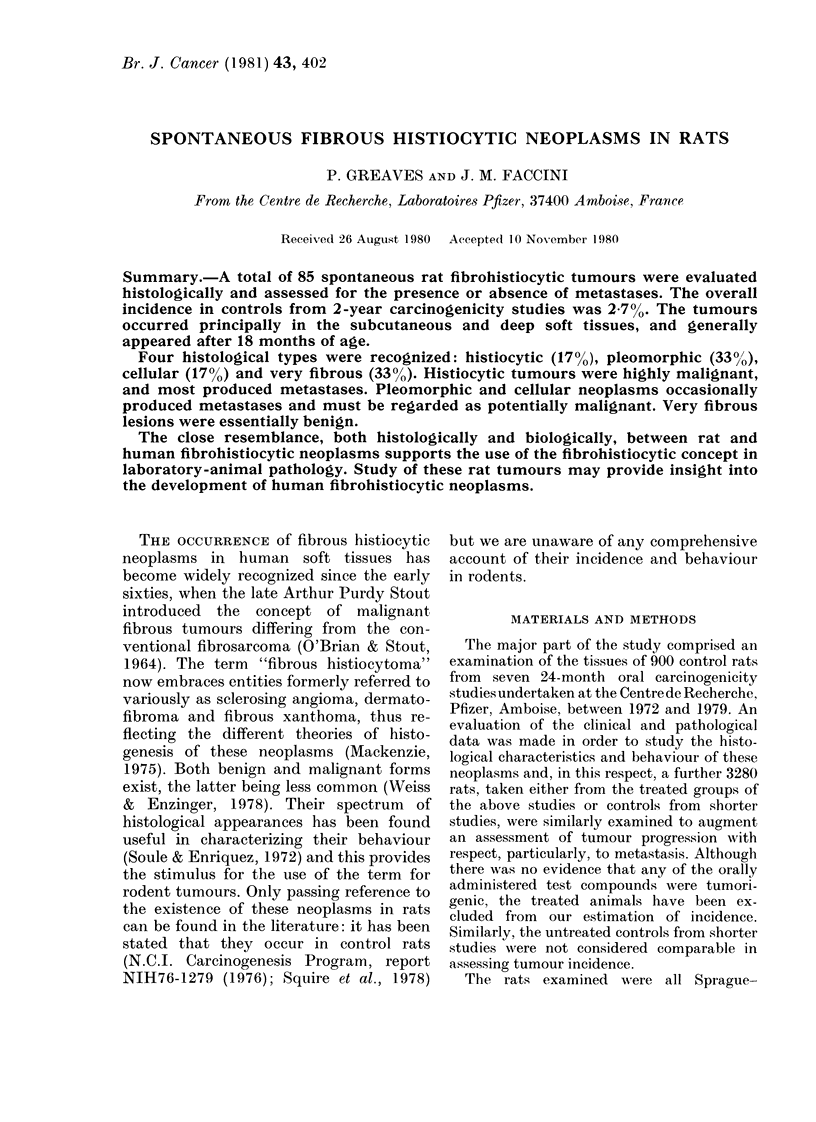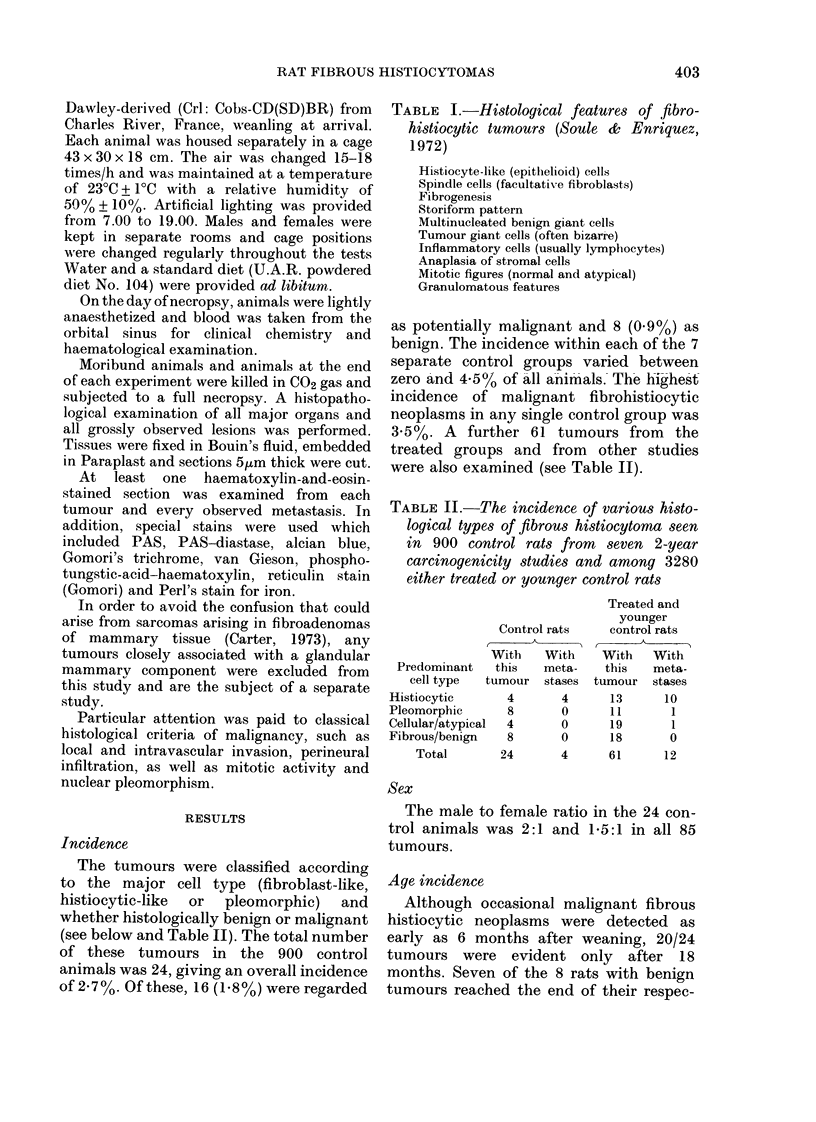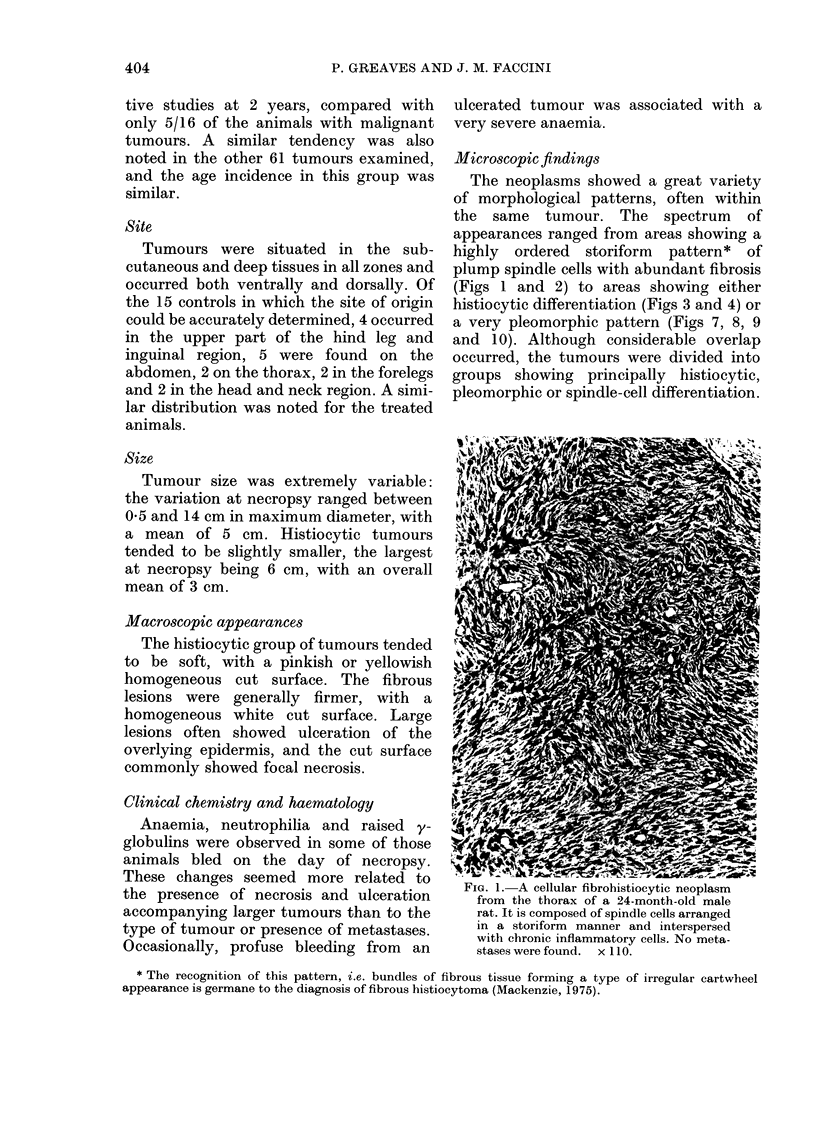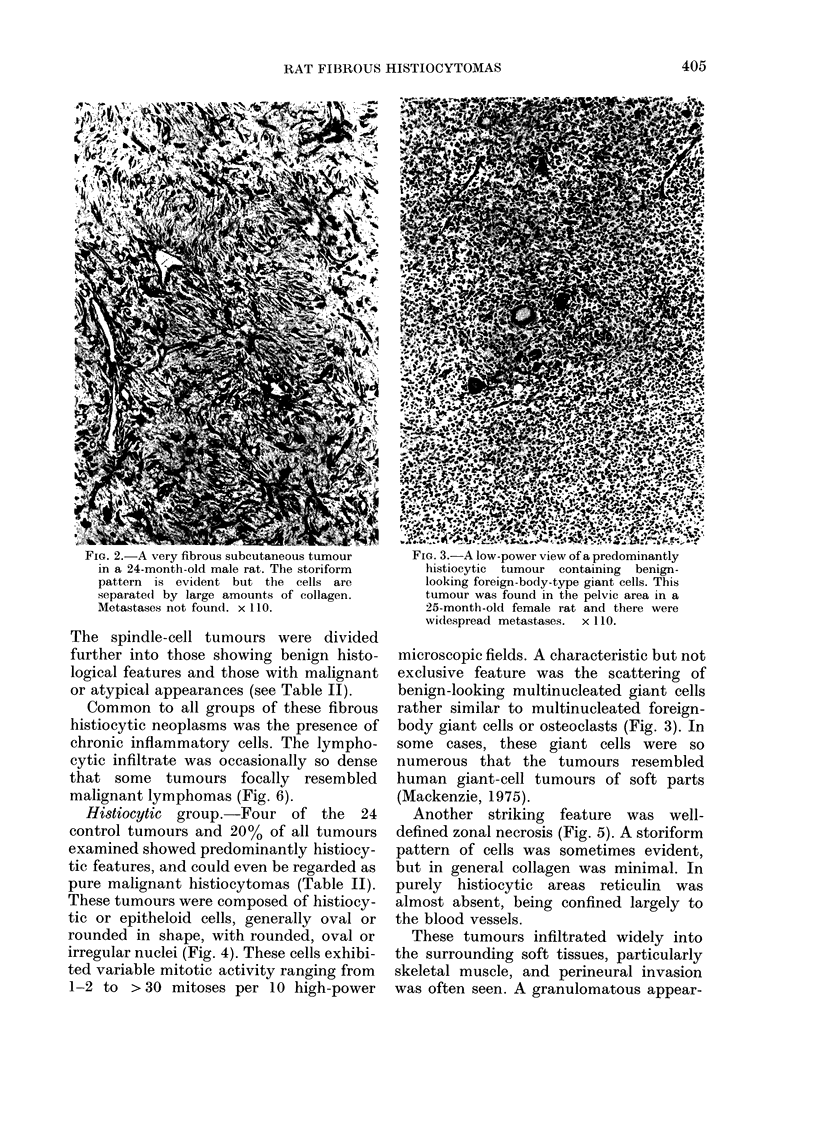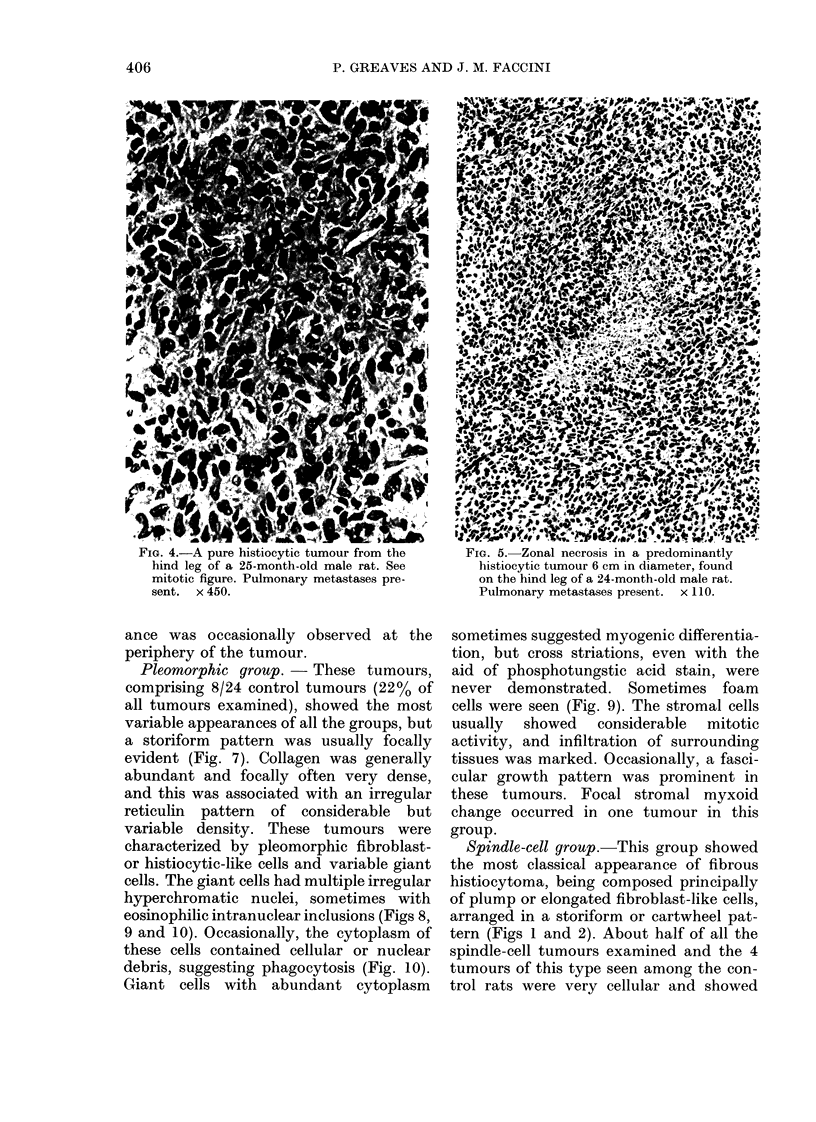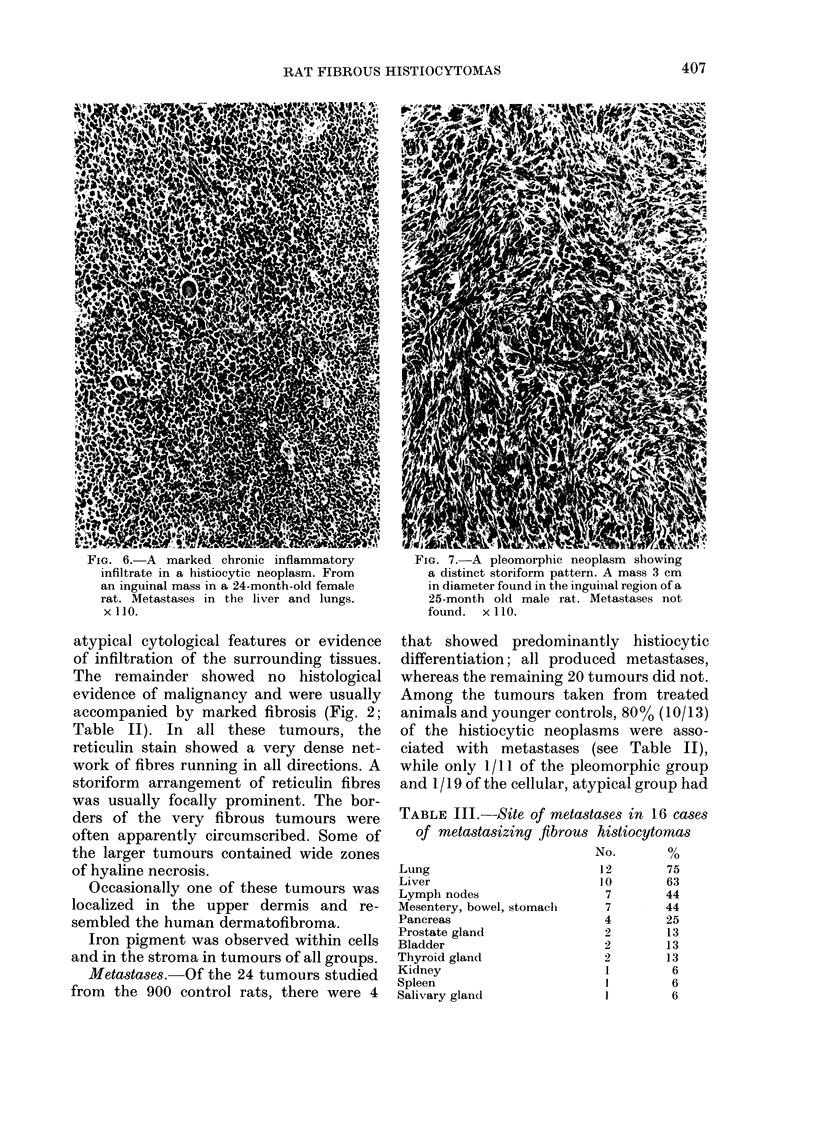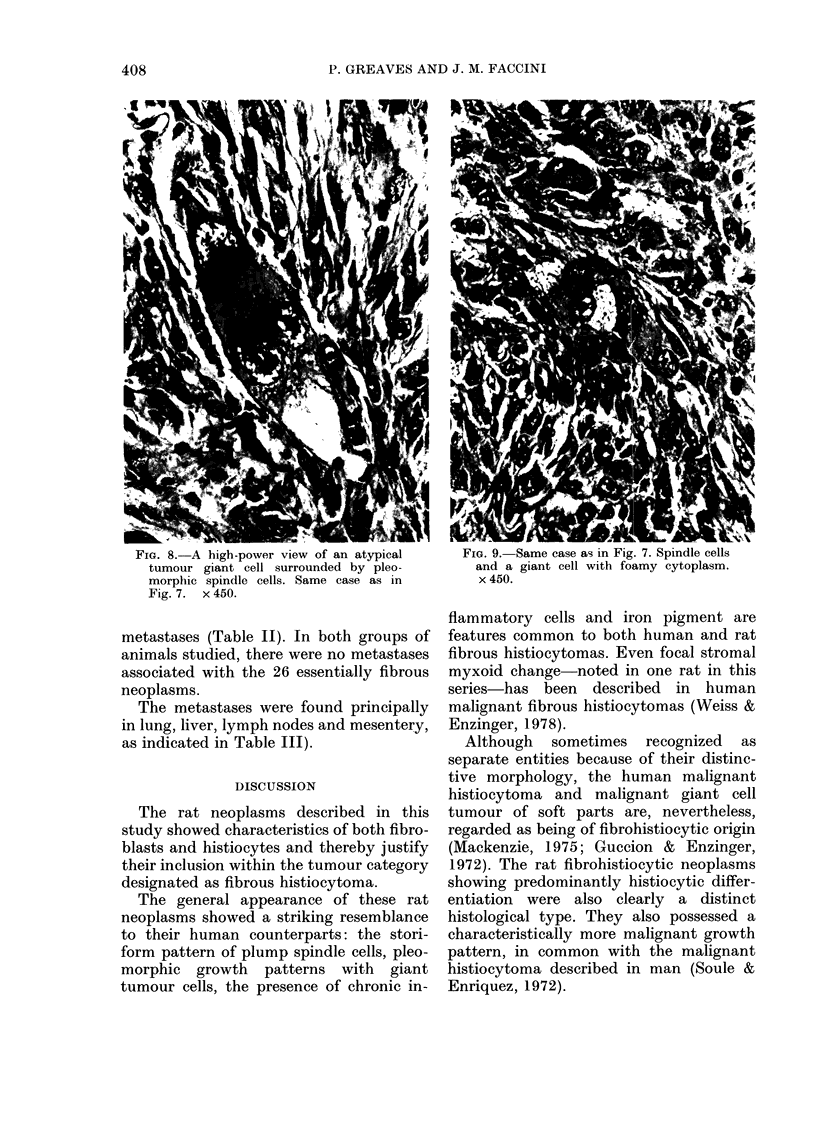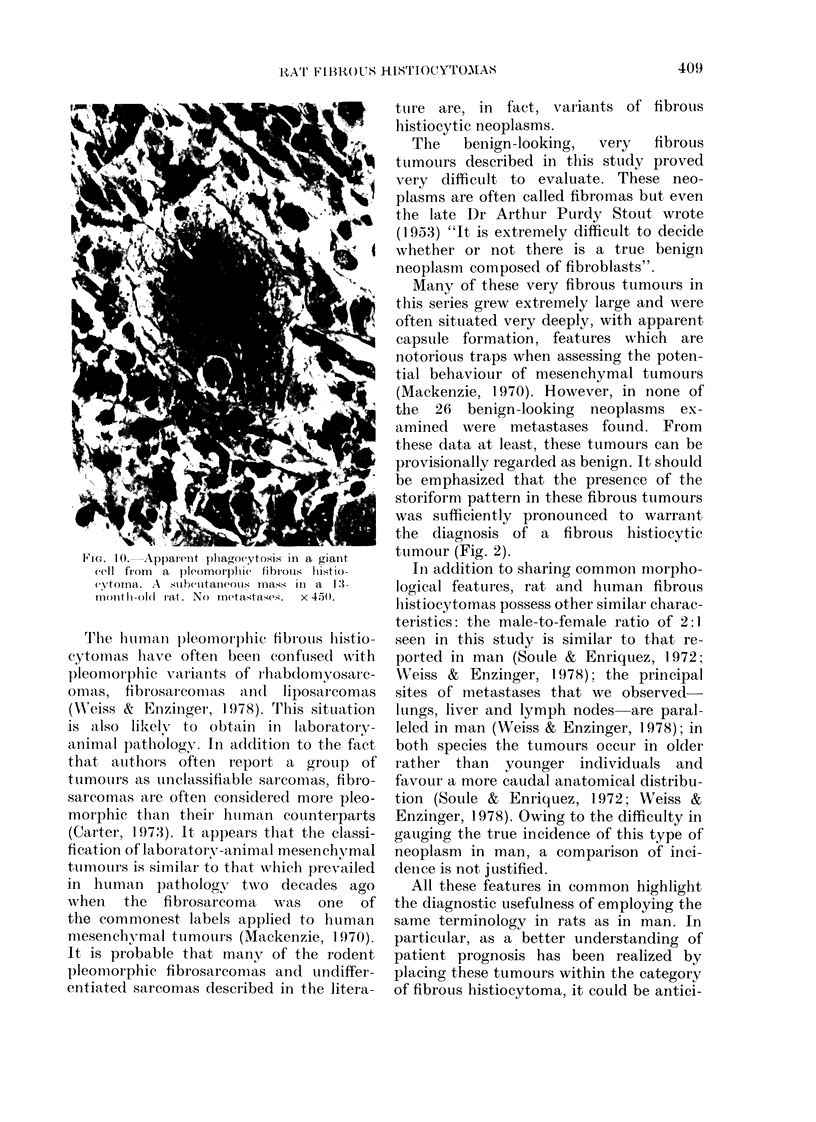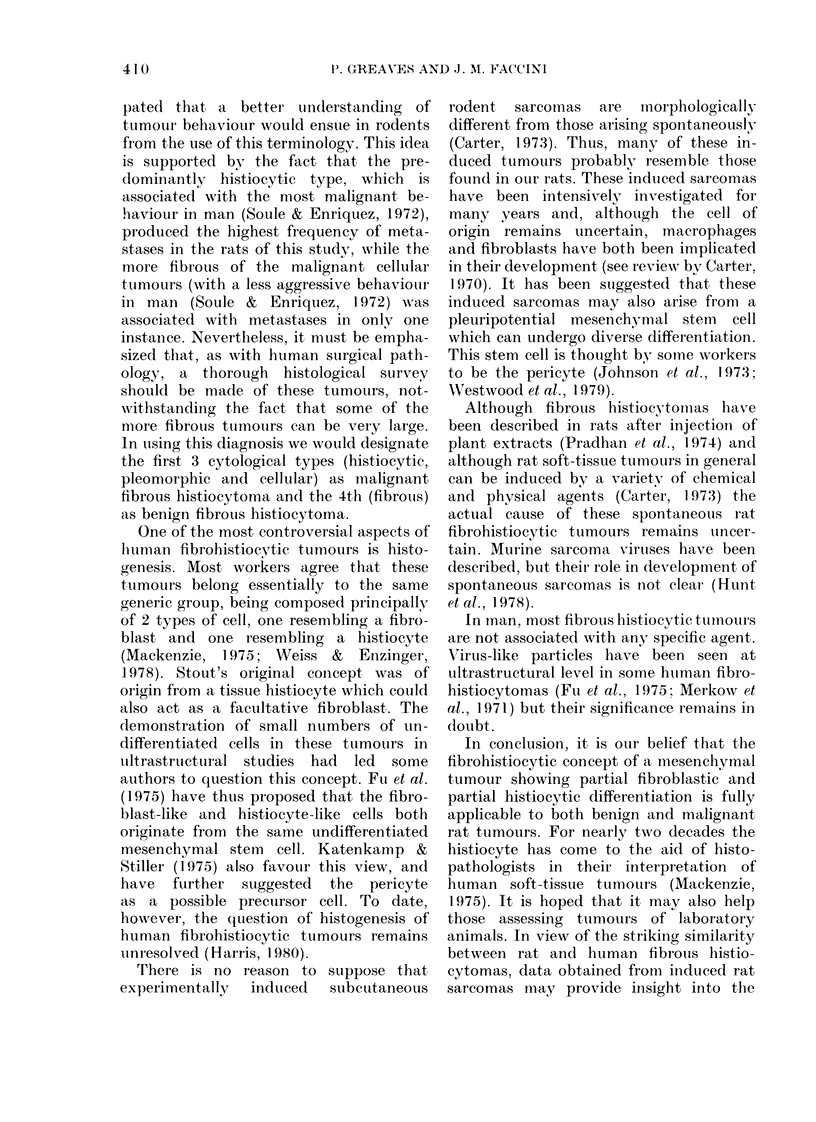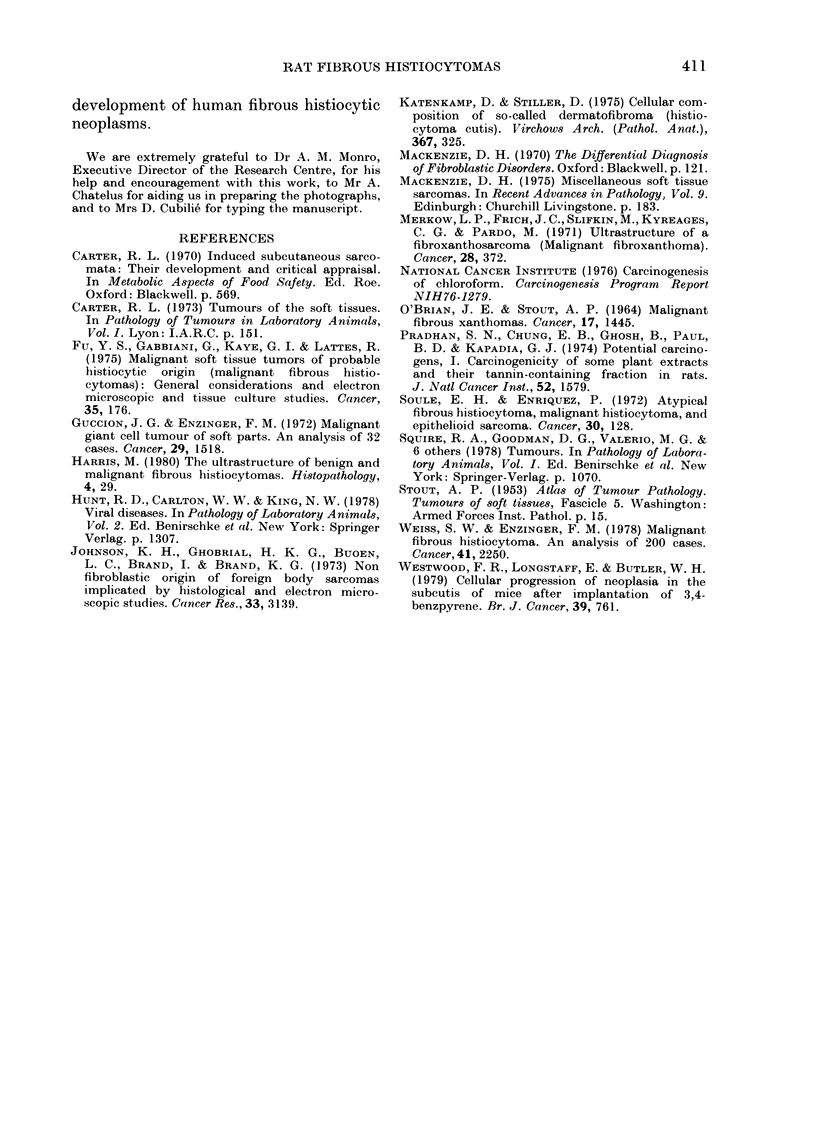# Spontaneous fibrous histiocytic neoplasms in rats.

**DOI:** 10.1038/bjc.1981.61

**Published:** 1981-03

**Authors:** P. Greaves, J. M. Faccini

## Abstract

**Images:**


*Br. J. Cancer* (1981) **43**, 402

### SPONTANEOUS FIBROUS HISTIOCYTIC NEOPLASMS IN RATS

P. GREAVES AND J. M. FACCINI

*From the Centre de Recherche, Laboratoires Pfizer,* 37400 *Amboise, France*

Received 26 August 1980 Accepted 10 November 1980

**Summary.—A total of 85 spontaneous rat fibrohistiocytic tumours were evaluated histologically and assessed for the presence or absence of metastases. The overall incidence in controls from 2-year carcinogenicity studies was 2·7%. The tumours occurred principally in the subcutaneous and deep soft tissues, and generally appeared after 18 months of age.**

**Four histological types were recognized: histiocytic (17%), pleomorphic (33%), cellular (17%) and very fibrous (33%). Histiocytic tumours were highly malignant, and most produced metastases. Pleomorphic and cellular neoplasms occasionally produced metastases and must be regarded as potentially malignant. Very fibrous lesions were essentially benign.**

**The close resemblance, both histologically and biologically, between rat and human fibrohistiocytic neoplasms supports the use of the fibrohistiocytic concept in laboratory-animal pathology. Study of these rat tumours may provide insight into the development of human fibrohistiocytic neoplasms.**

THE OCCURRENCE of fibrous histiocytic neoplasms in human soft tissues has become widely recognized since the early sixties, when the late Arthur Purdy Stout introduced the concept of malignant fibrous tumours differing from the conventional fibrosarcoma (O'Brian & Stout, 1964). The term "fibrous histiocytoma" now embraces entities formerly referred to variously as sclerosing angioma, dermatofibroma and fibrous xanthoma, thus reflecting the different theories of histogenesis of these neoplasms (Mackenzie, 1975). Both benign and malignant forms exist, the latter being less common (Weiss & Enzinger, 1978). Their spectrum of histological appearances has been found useful in characterizing their behaviour (Soule & Enriquez, 1972) and this provides the stimulus for the use of the term for rodent tumours. Only passing reference to the existence of these neoplasms in rats can be found in the literature: it has been stated that they occur in control rats (N.C.I. Carcinogenesis Program, report NIH76-1279 (1976); Squire *et al.*, 1978) but we are unaware of any comprehensive account of their incidence and behaviour in rodents.

### MATERIALS AND METHODS

The major part of the study comprised an examination of the tissues of 900 control rats from seven 24-month oral carcinogenicity studies undertaken at the Centre de Recherche, Pfizer, Amboise, between 1972 and 1979. An evaluation of the clinical and pathological data was made in order to study the histological characteristics and behaviour of these neoplasms and, in this respect, a further 3280 rats, taken either from the treated groups of the above studies or controls from shorter studies, were similarly examined to augment an assessment of tumour progression with respect, particularly, to metastasis. Although there was no evidence that any of the orally administered test compounds were tumorigenic, the treated animals have been excluded from our estimation of incidence. Similarly, the untreated controls from shorter studies were not considered comparable in assessing tumour incidence.

The rats examined were all Sprague–

Dawley-derived (Crl: Cobs-CD(SD)BR) from Charles River, France, weanling at arrival. Each animal was housed separately in a cage 43 × 30 × 18 cm. The air was changed 15–18 times/h and was maintained at a temperature of 23°C ± 1°C with a relative humidity of 50% ± 10%. Artificial lighting was provided from 7.00 to 19.00. Males and females were kept in separate rooms and cage positions were changed regularly throughout the tests Water and a standard diet (U.A.R. powdered diet No. 104) were provided *ad libitum.*

On the day of necropsy, animals were lightly anaesthetized and blood was taken from the orbital sinus for clinical chemistry and haematological examination.

Moribund animals and animals at the end of each experiment were killed in $CO_2$ gas and subjected to a full necropsy. A histopathological examination of all major organs and all grossly observed lesions was performed. Tissues were fixed in Bouin's fluid, embedded in Paraplast and sections 5$\mu$m thick were cut.

At least one haematoxylin-and-eosin-stained section was examined from each tumour and every observed metastasis. In addition, special stains were used which included PAS, PAS–diastase, alcian blue, Gomori's trichrome, van Gieson, phosphotungstic-acid–haematoxylin, reticulin stain (Gomori) and Perl's stain for iron.

In order to avoid the confusion that could arise from sarcomas arising in fibroadenomas of mammary tissue (Carter, 1973), any tumours closely associated with a glandular mammary component were excluded from this study and are the subject of a separate study.

Particular attention was paid to classical histological criteria of malignancy, such as local and intravascular invasion, perineural infiltration, as well as mitotic activity and nuclear pleomorphism.

### RESULTS

### *Incidence*

The tumours were classified according to the major cell type (fibroblast-like, histiocytic-like or pleomorphic) and whether histologically benign or malignant (see below and Table II). The total number of these tumours in the 900 control animals was 24, giving an overall incidence of 2·7%. Of these, 16 (1·8%) were regarded as potentially malignant and 8 (0·9%) as benign. The incidence within each of the 7 separate control groups varied between zero and 4·5% of all animals. The highest incidence of malignant fibrohistiocytic neoplasms in any single control group was 3·5%. A further 61 tumours from the treated groups and from other studies were also examined (see Table II).

TABLE I.—*Histological features of fibrohistiocytic tumours (Soule & Enriquez, 1972)*

Histiocyte-like (epithelioid) cells
Spindle cells (facultative fibroblasts)
Fibrogenesis
Storiform pattern
Multinucleated benign giant cells
Tumour giant cells (often bizarre)
Inflammatory cells (usually lymphocytes)
Anaplasia of stromal cells
Mitotic figures (normal and atypical)
Granulomatous features

TABLE II.—*The incidence of various histological types of fibrous histiocytoma seen in* 900 *control rats from seven* 2-*year carcinogenicity studies and among* 3280 *either treated or younger control rats*

| | Control rats | | Treated and younger control rats | |
|---|---|---|---|---|
| Predominant cell type | With this tumour | With metastases | With this tumour | With metastases |
| Histiocytic | 4 | 4 | 13 | 10 |
| Pleomorphic | 8 | 0 | 11 | 1 |
| Cellular/atypical | 4 | 0 | 19 | 1 |
| Fibrous/benign | 8 | 0 | 18 | 0 |
| Total | 24 | 4 | 61 | 12 |

### *Sex*

The male to female ratio in the 24 control animals was 2:1 and 1·5:1 in all 85 tumours.

### *Age incidence*

Although occasional malignant fibrous histiocytic neoplasms were detected as early as 6 months after weaning, 20/24 tumours were evident only after 18 months. Seven of the 8 rats with benign tumours reached the end of their respective studies at 2 years, compared with only 5/16 of the animals with malignant tumours. A similar tendency was also noted in the other 61 tumours examined, and the age incidence in this group was similar.

*Site*

Tumours were situated in the subcutaneous and deep tissues in all zones and occurred both ventrally and dorsally. Of the 15 controls in which the site of origin could be accurately determined, 4 occurred in the upper part of the hind leg and inguinal region, 5 were found on the abdomen, 2 on the thorax, 2 in the forelegs and 2 in the head and neck region. A similar distribution was noted for the treated animals.

*Size*

Tumour size was extremely variable: the variation at necropsy ranged between 0·5 and 14 cm in maximum diameter, with a mean of 5 cm. Histiocytic tumours tended to be slightly smaller, the largest at necropsy being 6 cm, with an overall mean of 3 cm.

*Macroscopic appearances*

The histiocytic group of tumours tended to be soft, with a pinkish or yellowish homogeneous cut surface. The fibrous lesions were generally firmer, with a homogeneous white cut surface. Large lesions often showed ulceration of the overlying epidermis, and the cut surface commonly showed focal necrosis.

*Clinical chemistry and haematology*

Anaemia, neutrophilia and raised $\gamma$-globulins were observed in some of those animals bled on the day of necropsy. These changes seemed more related to the presence of necrosis and ulceration accompanying larger tumours than to the type of tumour or presence of metastases. Occasionally, profuse bleeding from an ulcerated tumour was associated with a very severe anaemia.

*Microscopic findings*

The neoplasms showed a great variety of morphological patterns, often within the same tumour. The spectrum of appearances ranged from areas showing a highly ordered storiform pattern* of plump spindle cells with abundant fibrosis (Figs 1 and 2) to areas showing either histiocytic differentiation (Figs 3 and 4) or a very pleomorphic pattern (Figs 7, 8, 9 and 10). Although considerable overlap occurred, the tumours were divided into groups showing principally histiocytic, pleomorphic or spindle-cell differentiation.

FIG. 1.—A cellular fibrohistiocytic neoplasm from the thorax of a 24-month-old male rat. It is composed of spindle cells arranged in a storiform manner and interspersed with chronic inflammatory cells. No metastases were found. ×110.

* The recognition of this pattern, *i.e.* bundles of fibrous tissue forming a type of irregular cartwheel appearance is germane to the diagnosis of fibrous histiocytoma (Mackenzie, 1975).

FIG. 2.—A very fibrous subcutaneous tumour in a 24-month-old male rat. The storiform pattern is evident but the cells are separated by large amounts of collagen. Metastases not found. × 110.

FIG. 3.—A low-power view of a predominantly histiocytic tumour containing benign-looking foreign-body-type giant cells. This tumour was found in the pelvic area in a 25-month-old female rat and there were widespread metastases. × 110.

The spindle-cell tumours were divided further into those showing benign histological features and those with malignant or atypical appearances (see Table II).

Common to all groups of these fibrous histiocytic neoplasms was the presence of chronic inflammatory cells. The lymphocytic infiltrate was occasionally so dense that some tumours focally resembled malignant lymphomas (Fig. 6).

*Histiocytic* group.—Four of the 24 control tumours and 20% of all tumours examined showed predominantly histiocytic features, and could even be regarded as pure malignant histiocytomas (Table II). These tumours were composed of histiocytic or epitheloid cells, generally oval or rounded in shape, with rounded, oval or irregular nuclei (Fig. 4). These cells exhibited variable mitotic activity ranging from 1–2 to >30 mitoses per 10 high-power microscopic fields. A characteristic but not exclusive feature was the scattering of benign-looking multinucleated giant cells rather similar to multinucleated foreign-body giant cells or osteoclasts (Fig. 3). In some cases, these giant cells were so numerous that the tumours resembled human giant-cell tumours of soft parts (Mackenzie, 1975).

Another striking feature was well-defined zonal necrosis (Fig. 5). A storiform pattern of cells was sometimes evident, but in general collagen was minimal. In purely histiocytic areas reticulin was almost absent, being confined largely to the blood vessels.

These tumours infiltrated widely into the surrounding soft tissues, particularly skeletal muscle, and perineural invasion was often seen. A granulomatous appear-

FIG. 4.—A pure histiocytic tumour from the hind leg of a 25-month-old male rat. See mitotic figure. Pulmonary metastases present. ×450.

FIG. 5.—Zonal necrosis in a predominantly histiocytic tumour 6 cm in diameter, found on the hind leg of a 24-month-old male rat. Pulmonary metastases present. ×110.

ance was occasionally observed at the periphery of the tumour.

*Pleomorphic group.* — These tumours, comprising 8/24 control tumours (22% of all tumours examined), showed the most variable appearances of all the groups, but a storiform pattern was usually focally evident (Fig. 7). Collagen was generally abundant and focally often very dense, and this was associated with an irregular reticulin pattern of considerable but variable density. These tumours were characterized by pleomorphic fibroblast- or histiocytic-like cells and variable giant cells. The giant cells had multiple irregular hyperchromatic nuclei, sometimes with eosinophilic intranuclear inclusions (Figs 8, 9 and 10). Occasionally, the cytoplasm of these cells contained cellular or nuclear debris, suggesting phagocytosis (Fig. 10). Giant cells with abundant cytoplasm sometimes suggested myogenic differentiation, but cross striations, even with the aid of phosphotungstic acid stain, were never demonstrated. Sometimes foam cells were seen (Fig. 9). The stromal cells usually showed considerable mitotic activity, and infiltration of surrounding tissues was marked. Occasionally, a fascicular growth pattern was prominent in these tumours. Focal stromal myxoid change occurred in one tumour in this group.

*Spindle-cell group.*—This group showed the most classical appearance of fibrous histiocytoma, being composed principally of plump or elongated fibroblast-like cells, arranged in a storiform or cartwheel pattern (Figs 1 and 2). About half of all the spindle-cell tumours examined and the 4 tumours of this type seen among the control rats were very cellular and showed

FIG. 6.—A marked chronic inflammatory infiltrate in a histiocytic neoplasm. From an inguinal mass in a 24-month-old female rat. Metastases in the liver and lungs. ×110.

FIG. 7.—A pleomorphic neoplasm showing a distinct storiform pattern. A mass 3 cm in diameter found in the inguinal region of a 25-month old male rat. Metastases not found. ×110.

atypical cytological features or evidence of infiltration of the surrounding tissues. The remainder showed no histological evidence of malignancy and were usually accompanied by marked fibrosis (Fig. 2; Table II). In all these tumours, the reticulin stain showed a very dense network of fibres running in all directions. A storiform arrangement of reticulin fibres was usually focally prominent. The borders of the very fibrous tumours were often apparently circumscribed. Some of the larger tumours contained wide zones of hyaline necrosis.

Occasionally one of these tumours was localized in the upper dermis and resembled the human dermatofibroma.

Iron pigment was observed within cells and in the stroma in tumours of all groups.

*Metastases*.—Of the 24 tumours studied from the 900 control rats, there were 4 that showed predominantly histiocytic differentiation; all produced metastases, whereas the remaining 20 tumours did not. Among the tumours taken from treated animals and younger controls, 80% (10/13) of the histiocytic neoplasms were associated with metastases (see Table II), while only 1/11 of the pleomorphic group and 1/19 of the cellular, atypical group had

TABLE III.—*Site of metastases in* 16 *cases of metastasizing fibrous histiocytomas*

| | No. | % |
|---|---|---|
| Lung | 12 | 75 |
| Liver | 10 | 63 |
| Lymph nodes | 7 | 44 |
| Mesentery, bowel, stomach | 7 | 44 |
| Pancreas | 4 | 25 |
| Prostate gland | 2 | 13 |
| Bladder | 2 | 13 |
| Thyroid gland | 2 | 13 |
| Kidney | 1 | 6 |
| Spleen | 1 | 6 |
| Salivary gland | 1 | 6 |

FIG. 8.—A high-power view of an atypical tumour giant cell surrounded by pleomorphic spindle cells. Same case as in Fig. 7. ×450.

FIG. 9.—Same case as in Fig. 7. Spindle cells and a giant cell with foamy cytoplasm. ×450.

metastases (Table II). In both groups of animals studied, there were no metastases associated with the 26 essentially fibrous neoplasms.

The metastases were found principally in lung, liver, lymph nodes and mesentery, as indicated in Table III).

### DISCUSSION

The rat neoplasms described in this study showed characteristics of both fibroblasts and histiocytes and thereby justify their inclusion within the tumour category designated as fibrous histiocytoma.

The general appearance of these rat neoplasms showed a striking resemblance to their human counterparts: the storiform pattern of plump spindle cells, pleomorphic growth patterns with giant tumour cells, the presence of chronic inflammatory cells and iron pigment are features common to both human and rat fibrous histiocytomas. Even focal stromal myxoid change—noted in one rat in this series—has been described in human malignant fibrous histiocytomas (Weiss & Enzinger, 1978).

Although sometimes recognized as separate entities because of their distinctive morphology, the human malignant histiocytoma and malignant giant cell tumour of soft parts are, nevertheless, regarded as being of fibrohistiocytic origin (Mackenzie, 1975; Guccion & Enzinger, 1972). The rat fibrohistiocytic neoplasms showing predominantly histiocytic differentiation were also clearly a distinct histological type. They also possessed a characteristically more malignant growth pattern, in common with the malignant histiocytoma described in man (Soule & Enriquez, 1972).

FIG. 10.—Apparent phagocytosis in a giant cell from a pleomorphic fibrous histiocytoma. A subcutaneous mass in a 13-month-old rat. No metastases. ×450.

The human pleomorphic fibrous histiocytomas have often been confused with pleomorphic variants of rhabdomyosarcomas, fibrosarcomas and liposarcomas (Weiss & Enzinger, 1978). This situation is also likely to obtain in laboratory-animal pathology. In addition to the fact that authors often report a group of tumours as unclassifiable sarcomas, fibrosarcomas are often considered more pleomorphic than their human counterparts (Carter, 1973). It appears that the classification of laboratory-animal mesenchymal tumours is similar to that which prevailed in human pathology two decades ago when the fibrosarcoma was one of the commonest labels applied to human mesenchymal tumours (Mackenzie, 1970). It is probable that many of the rodent pleomorphic fibrosarcomas and undifferentiated sarcomas described in the literature are, in fact, variants of fibrous histiocytic neoplasms.

The benign-looking, very fibrous tumours described in this study proved very difficult to evaluate. These neoplasms are often called fibromas but even the late Dr Arthur Purdy Stout wrote (1953) "It is extremely difficult to decide whether or not there is a true benign neoplasm composed of fibroblasts".

Many of these very fibrous tumours in this series grew extremely large and were often situated very deeply, with apparent capsule formation, features which are notorious traps when assessing the potential behaviour of mesenchymal tumours (Mackenzie, 1970). However, in none of the 26 benign-looking neoplasms examined were metastases found. From these data at least, these tumours can be provisionally regarded as benign. It should be emphasized that the presence of the storiform pattern in these fibrous tumours was sufficiently pronounced to warrant the diagnosis of a fibrous histiocytic tumour (Fig. 2).

In addition to sharing common morphological features, rat and human fibrous histiocytomas possess other similar characteristics: the male-to-female ratio of 2:1 seen in this study is similar to that reported in man (Soule & Enriquez, 1972; Weiss & Enzinger, 1978); the principal sites of metastases that we observed—lungs, liver and lymph nodes—are paralleled in man (Weiss & Enzinger, 1978); in both species the tumours occur in older rather than younger individuals and favour a more caudal anatomical distribution (Soule & Enriquez, 1972; Weiss & Enzinger, 1978). Owing to the difficulty in gauging the true incidence of this type of neoplasm in man, a comparison of incidence is not justified.

All these features in common highlight the diagnostic usefulness of employing the same terminology in rats as in man. In particular, as a better understanding of patient prognosis has been realized by placing these tumours within the category of fibrous histiocytoma, it could be anticipated that a better understanding of tumour behaviour would ensue in rodents from the use of this terminology. This idea is supported by the fact that the predominantly histiocytic type, which is associated with the most malignant behaviour in man (Soule & Enriquez, 1972), produced the highest frequency of metastases in the rats of this study, while the more fibrous of the malignant cellular tumours (with a less aggressive behaviour in man (Soule & Enriquez, 1972) was associated with metastases in only one instance. Nevertheless, it must be emphasized that, as with human surgical pathology, a thorough histological survey should be made of these tumours, notwithstanding the fact that some of the more fibrous tumours can be very large. In using this diagnosis we would designate the first 3 cytological types (histiocytic, pleomorphic and cellular) as malignant fibrous histiocytoma and the 4th (fibrous) as benign fibrous histiocytoma.

One of the most controversial aspects of human fibrohistiocytic tumours is histogenesis. Most workers agree that these tumours belong essentially to the same generic group, being composed principally of 2 types of cell, one resembling a fibroblast and one resembling a histiocyte (Mackenzie, 1975; Weiss & Enzinger, 1978). Stout's original concept was of origin from a tissue histiocyte which could also act as a facultative fibroblast. The demonstration of small numbers of undifferentiated cells in these tumours in ultrastructural studies had led some authors to question this concept. Fu *et al.* (1975) have thus proposed that the fibroblast-like and histiocyte-like cells both originate from the same undifferentiated mesenchymal stem cell. Katenkamp & Stiller (1975) also favour this view, and have further suggested the pericyte as a possible precursor cell. To date, however, the question of histogenesis of human fibrohistiocytic tumours remains unresolved (Harris, 1980).

There is no reason to suppose that experimentally induced subcutaneous rodent sarcomas are morphologically different from those arising spontaneously (Carter, 1973). Thus, many of these induced tumours probably resemble those found in our rats. These induced sarcomas have been intensively investigated for many years and, although the cell of origin remains uncertain, macrophages and fibroblasts have both been implicated in their development (see review by Carter, 1970). It has been suggested that these induced sarcomas may also arise from a pleuripotential mesenchymal stem cell which can undergo diverse differentiation. This stem cell is thought by some workers to be the pericyte (Johnson *et al.*, 1973; Westwood *et al.*, 1979).

Although fibrous histiocytomas have been described in rats after injection of plant extracts (Pradhan *et al.*, 1974) and although rat soft-tissue tumours in general can be induced by a variety of chemical and physical agents (Carter, 1973) the actual cause of these spontaneous rat fibrohistiocytic tumours remains uncertain. Murine sarcoma viruses have been described, but their role in development of spontaneous sarcomas is not clear (Hunt *et al.*, 1978).

In man, most fibrous histiocytic tumours are not associated with any specific agent. Virus-like particles have been seen at ultrastructural level in some human fibrohistiocytomas (Fu *et al.*, 1975; Merkow *et al.*, 1971) but their significance remains in doubt.

In conclusion, it is our belief that the fibrohistiocytic concept of a mesenchymal tumour showing partial fibroblastic and partial histiocytic differentiation is fully applicable to both benign and malignant rat tumours. For nearly two decades the histiocyte has come to the aid of histopathologists in their interpretation of human soft-tissue tumours (Mackenzie, 1975). It is hoped that it may also help those assessing tumours of laboratory animals. In view of the striking similarity between rat and human fibrous histiocytomas, data obtained from induced rat sarcomas may provide insight into the development of human fibrous histiocytic neoplasms.

We are extremely grateful to Dr A. M. Monro, Executive Director of the Research Centre, for his help and encouragement with this work, to Mr A. Chatelus for aiding us in preparing the photographs, and to Mrs D. Cubilié for typing the manuscript.

### REFERENCES

CARTER, R. L. (1970) Induced subcutaneous sarcomata: Their development and critical appraisal. In *Metabolic Aspects of Food Safety*. Ed. Roe. Oxford: Blackwell. p. 569.

CARTER, R. L. (1973) Tumours of the soft tissues. In *Pathology of Tumours in Laboratory Animals, Vol. I*. Lyon: I.A.R.C. p. 151.

FU, Y. S., GABBIANI, G., KAYE, G. I. & LATTES, R. (1975) Malignant soft tissue tumors of probable histiocytic origin (malignant fibrous histiocytomas): General considerations and electron microscopic and tissue culture studies. *Cancer*, **35**, 176.

GUCCION, J. G. & ENZINGER, F. M. (1972) Malignant giant cell tumour of soft parts. An analysis of 32 cases. *Cancer*, **29**, 1518.

HARRIS, M. (1980) The ultrastructure of benign and malignant fibrous histiocytomas. *Histopathology*, **4**, 29.

HUNT, R. D., CARLTON, W. W. & KING, N. W. (1978) Viral diseases. In *Pathology of Laboratory Animals, Vol. 2*. Ed. Benirschke *et al.* New York: Springer Verlag. p. 1307.

JOHNSON, K. H., GHOBRIAL, H. K. G., BUOEN, L. C., BRAND, I. & BRAND, K. G. (1973) Non fibroblastic origin of foreign body sarcomas implicated by histological and electron microscopic studies. *Cancer Res.*, **33**, 3139.

KATENKAMP, D. & STILLER, D. (1975) Cellular composition of so-called dermatofibroma (histiocytoma cutis). *Virchows Arch.* (*Pathol. Anat.*), **367**, 325.

MACKENZIE, D. H. (1970) *The Differential Diagnosis of Fibroblastic Disorders*. Oxford: Blackwell. p. 121.

MACKENZIE, D. H. (1975) Miscellaneous soft tissue sarcomas. In *Recent Advances in Pathology, Vol. 9*. Edinburgh: Churchill Livingstone. p. 183.

MERKOW, L. P., FRICH, J. C., SLIFKIN, M., KYREAGES, C. G. & PARDO, M. (1971) Ultrastructure of a fibroxanthosarcoma (Malignant fibroxanthoma). *Cancer*, **28**, 372.

NATIONAL CANCER INSTITUTE (1976) Carcinogenesis of chloroform. *Carcinogenesis Program Report NIH76-1279*.

O'BRIAN, J. E. & STOUT, A. P. (1964) Malignant fibrous xanthomas. *Cancer*, **17**, 1445.

PRADHAN, S. N., CHUNG, E. B., GHOSH, B., PAUL, B. D. & KAPADIA, G. J. (1974) Potential carcinogens, I. Carcinogenicity of some plant extracts and their tannin-containing fraction in rats. *J. Natl Cancer Inst.*, **52**, 1579.

SOULE, E. H. & ENRIQUEZ, P. (1972) Atypical fibrous histiocytoma, malignant histiocytoma, and epithelioid sarcoma. *Cancer*, **30**, 128.

SQUIRE, R. A., GOODMAN, D. G., VALERIO, M. G. & 6 others (1978) Tumours. In *Pathology of Laboratory Animals, Vol. I*. Ed. Benirschke *et al.* New York: Springer-Verlag. p. 1070.

STOUT, A. P. (1953) *Atlas of Tumour Pathology. Tumours of soft tissues*, Fascicle 5. Washington: Armed Forces Inst. Pathol. p. 15.

WEISS, S. W. & ENZINGER, F. M. (1978) Malignant fibrous histiocytoma. An analysis of 200 cases. *Cancer*, **41**, 2250.

WESTWOOD, F. R., LONGSTAFF, E. & BUTLER, W. H. (1979) Cellular progression of neoplasia in the subcutis of mice after implantation of 3,4-benzpyrene. *Br. J. Cancer*, **39**, 761.